# Accuracy of 3D Virtual Surgical Planning Compared to the Traditional Two-Dimensional Method in Orthognathic Surgery: A Literature Review

**DOI:** 10.7759/cureus.73477

**Published:** 2024-11-11

**Authors:** Mohammed Mahmoud Shalabi, Khaldoun M.A. Darwich, Mohammad N. Kheshfeh, Mohammad Y. Hajeer

**Affiliations:** 1 Department of Oral and Maxillofacial Surgery, Faculty of Dentistry, University of Damascus, Damascus, SYR; 2 Department of Orthodontics, Faculty of Dentistry, University of Damascus, Damascus, SYR

**Keywords:** 3d printing in orthognathic surgery, accuracy of prediction for hard tissue changes, accuracy of prediction for soft tissue changes, orthognathic surgery, surgical guides, surgical prediction, traditional surgical planning, virtual orthognathic patient, virtual surgical planning

## Abstract

With the innovation of three-dimensional imaging and printing techniques, computer-aided surgical planning, also known as virtual surgical planning (VSP), has revolutionized orthognathic surgery. Designing and manufacturing patient-specific surgical guides using three-dimensional printing techniques to improve surgical outcomes is now possible. This article presents an overview of VSP in orthognathic surgery and discusses the advantages and accuracy of this technique compared to traditional surgical planning (TSP). A PubMed and Google Scholar search was conducted to find relevant articles published over the past 10 years. The search revealed 2,581 articles, of which 36 full-text articles specifically addressed the topic of this study. The review concludes that VSP in orthognathic surgery provides optimal functional and aesthetic results, enhances patient satisfaction, ensures precise translation of the treatment plan, and facilitates intraoperative manipulation.

## Introduction and background

Orthognathic surgery, a branch of oral and maxillofacial surgery, addresses dentofacial deformities in patients with various types of malocclusion [1]. The primary goals of orthognathic surgery are to enhance chewing activity, breathing, and speaking capabilities and improve facial aesthetics [2,3]. Successful orthognathic surgery necessitates close cooperation between the surgeon and orthodontist, with the orthodontist managing pre- and postoperative orthodontics to achieve correct occlusion for both functional and cosmetic outcomes [4]. Accurate treatment planning is crucial for successful orthognathic surgery. A thorough diagnostic protocol is essential to simulate surgical procedures and predict orthodontic movements, ultimately establishing precise occlusion [5]. Treatment planning and presurgical protocols, including the tooth-jaw relationship, osteotomy site, and surgical procedure, are key to positive surgical outcomes [6]. Traditional surgical planning (TSP) for orthognathic surgery involves clinical and physical examinations, photographs, two-dimensional cephalometric radiographs, model analysis, and a face bow with a semi-adjustable articulator [7]. However, the multiple steps involved in TSP can lead to cumulative errors and are time-consuming [8].

Recent advancements, such as cone-beam computed tomography (CBCT) scanning and three-dimensional surface technology with computer-aided design software, have revolutionized orthognathic surgery [9]. These technologies facilitate virtual orthognathic planning (VOP) by collecting CBCT data and analyzing digital dental models. The position of the dental arch and each tooth can be evaluated concerning the patient's facial and cranial anatomy [5]. Furthermore, three-dimensional data, cephalometric measurements, and virtual planning for surgical procedures enhance the prediction of the correlation between dentoskeletal complex movements and soft tissue response [10,11]. Virtual surgical planning (VSP) encompasses several steps, including data collection, pre-planning sessions, planning, and manufacturing surgical splints to ensure accurate diagnosis and treatment [7].

Recent studies have highlighted the importance of virtual 3D prediction in achieving positive results in orthognathic surgery. Stokbro et al. summarized five clinical cases, concluding that the VSP approach demonstrated strong predictability and reproducibility for jaw surgery [12]. However, due to the absence of clinical control, it was impossible to determine which planning strategy was superior. Gaber et al. conducted a systematic review to evaluate the accuracy of VSP and proposed a universal protocol with a minimal margin of error, including automated or semi-automated evaluation, inter- and intra-observer reliability, and voxel registration on the cranial base [13]. There is a strong likelihood that VSP will eventually replace TSP in orthognathic surgery.

Nevertheless, researchers question whether the current VSP technique is more accurate than the TSP technique [14]. Since 2003, various studies have compared the accuracy, time, cost, and patient satisfaction of TSP and VSP [15]. A recent systematic review by Nilsson et al. [16] indicated that, compared to traditional methods, computer-assisted design reduced surgical and ischemia times for maxillofacial reconstruction and shortened preoperative planning times for orthognathic surgery [16]. However, further research is necessary to determine whether VSP is superior to TSP in all aspects. This literature review aims to evaluate the accuracy of VSP compared with TSP in orthognathic surgery by highlighting the advantages and limitations of each technique.

## Review

The search strategy

A comprehensive literature review was conducted using PubMed® and Google™ Scholar to identify relevant English-language articles published since 2013. Specific search terms included “accuracy,” “comparing,” “virtual surgical planning,” “conventional surgical planning,” “traditional surgical planning,” and “orthognathic surgery.” The search strategy was designed to gather a comprehensive collection of studies addressing key aspects of surgical planning in orthognathic procedures. PubMed and Google Scholar were selected for their extensive databases and access to a wide range of peer-reviewed articles.

The inclusion criteria were specifically targeted toward studies that provided quantitative data on the accuracy of surgical outcomes, comparisons between VSP and traditional methods, and clinical evaluations of orthognathic surgery results. The search process involved several steps: 1) Initial Search: The initial search used the specified keywords to identify a broad range of articles. 2) Screening: Titles and abstracts of identified articles were screened to exclude irrelevant studies, non-English publications, and articles published before 2013. 3) Full-Text Review: Full texts of the remaining articles were reviewed to ensure they met the inclusion criteria. Studies that provided detailed comparisons between VSP and TSP methods and reported on the accuracy of surgical outcomes were included. 4) Information Extraction: Relevant data were extracted from the selected articles, including methods of surgical planning, characteristics and techniques, accuracy measurements, surgical accuracy, and clinical outcomes (hard and soft tissue accuracy).

Search results

A comprehensive search yielded 2,850 articles. After excluding systematic reviews, pilot studies, and duplicate articles, 135 articles remained. From these, 36 full-text articles were selected for detailed analysis.

Summary of studies regarding traditional and 3D-based treatment planning

Orthognathic Surgery

Orthognathic surgery is a standard treatment to correct severe malocclusion or facial asymmetry [17]. If these cases are not treated, unwanted effects may occur on oral and emotional health [18]. During this surgical procedure, an osteotomy of the mandible and maxilla is performed, and the mandible and maxilla are moved into a new position and fixed with metal braces [19]. The most important step in orthognathic surgery is surgical planning, which is considered challenging. An accurate diagnostic protocol is needed to predict orthodontic movement and simulate surgical movements to obtain a stable occlusion after the surgery [5]. Hence, orthognathic surgical planning and presurgical procedures are crucial to the operation's success, including the osteotomy site, tooth-jaw relationship, and surgical approach [7].

Traditional Orthognathic Surgery Treatment Planning

**Figure 1:**
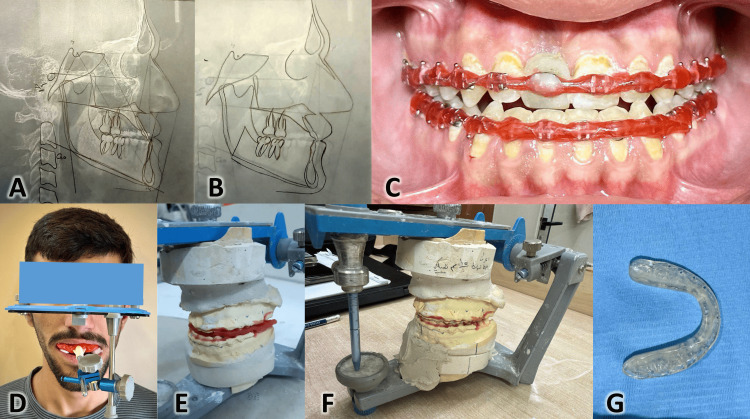
The traditional two-dimensional method of orthognathic surgery planning. A) Tracing of the lateral cephalometric image of the patient before surgery; B) The cephalometric prediction after performing the required movements of the bony segments; C) Preparation of the dental arches before taking impressions by covering the vestibular surfaces of teeth and brackets with wax; D) Face bow registration of the relationship between the maxilla and the craniofacial complex; E) Impression are poured and plaster models are mounted on a semi-adjustable articulator; F) Simulation of the orthognathic movements are performed on the plaster models with specific amounts of movements to arrive at good occlusion; G) The surgical splints (or wafers) are constructed manually using acrylic materials to be used intra-orally as surgical guides.

Orthognathic surgery is considered an ideal treatment choice for severe skeletal malocclusion cases. Traditionally, orthognathic surgery planning relies on preoperative procedures, including making dental casts, face bow registration, two-dimensional radiographic imaging such as panoramic and cephalometric imaging, simulation of the treatment outcome, and the fabrication of the surgical occlusal wafers [3], as shown in Figure 1. However, there are many limitations to this conventional method. For example, 2D imaging cannot truly represent three-dimensional details, and physical models cannot simulate the dynamic nature of facial structures and movement [20].

A full traditional presurgical diagnosis includes cephalometric tracing, model making, and face bow registration. Following this, model mounting, surgery, and splint fabrication should be completed [21]. Conventional techniques in orthognathic surgery involve surgical osteotomies and bone grafting, depending on the patient’s case. Despite their proven efficacy, these procedures can be very aggressive, and many postsurgical complications may occur due to the lack of accuracy in the presurgical plan [22].

Traditional surgery planning is time-consuming and involves potential errors due to its multiple steps. Some complications related to conventional methods include bleeding, infection, wound healing problems, nerve damage, and relapse. Sometimes, severe complications may necessitate a second surgical intervention [23]. From the patient’s viewpoint, there are disadvantages to TSP, such as long healing times, invasive procedures, and dissatisfaction with the results, as this method does not allow patients to predict and visualize the surgical outcomes [2]. Enhancing the predictability and simplicity of orthognathic surgery planning could be a major improvement for both surgeons and orthodontists [21].

Virtual Surgical Planning in Orthognathic Surgery

VSP utilizes digital data for operative procedures, representing a significant advancement in the field of orthognathic and oral maxillofacial surgery. The process begins with collecting patient imaging data using CT or CBCT scans [2]. These images are then converted into three-dimensional digital models through software algorithms, which can be manipulated to simulate surgical outcomes and create patient-specific surgical guides [22]. This technology enables the visualization of anatomical structures and facilitates virtual osteotomy and remodeling [24]. VSP for orthognathic surgery involves a multidisciplinary team of surgeons, orthodontists, and technicians. Landmarks are initially identified on bone surfaces and soft tissues [25]. The distances between these landmarks are evaluated and compared to optimal reference values to determine the treatment goals [26].

Virtual Surgery Planning Procedure

**Figure 2:**
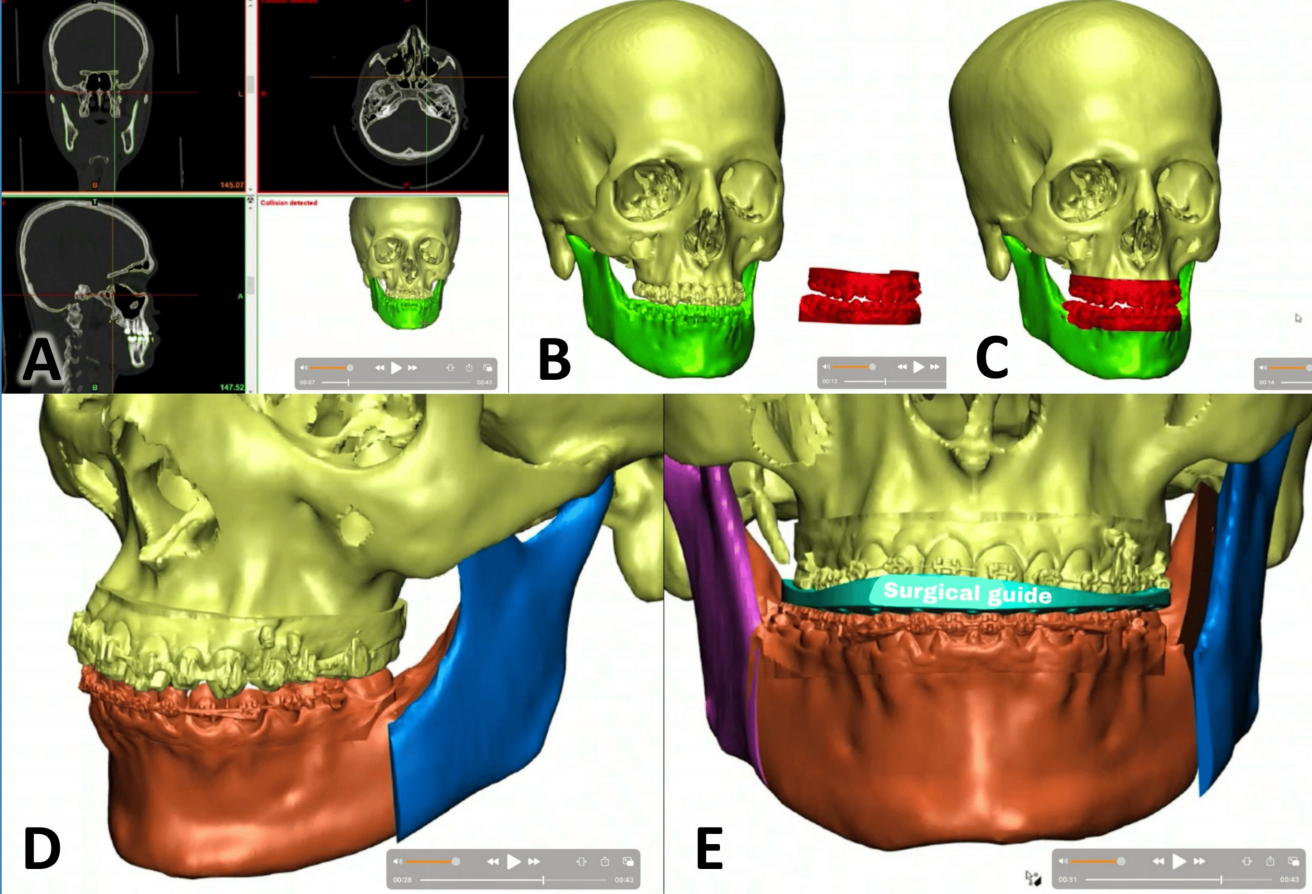
The stages of the virtual surgical planning (VSP) in orthognathic surgery using 3D patient records. A) A cone-beam computed tomography (CBCT) scan of the face is performed, including axial, sagittal, coronal, and 3D rendered views; B) the scanning of the teeth with an intraoral 3D camera before transferring the data to the 3D model of the skull; C) the 3D scan is transferred to the radiographic model, involving a matching procedure where the teeth and parts of the bone on the model are replaced with the precise jaw relationships obtained from the 3D scan; D) the virtual 3D planning of the surgical interventions according to the planned movements of one or both jaws; E) a surgical guide is fabricated to accurately transfer the newly established jaw relationship for implementation during surgery.

Establishing VSP involves many steps before any surgical intervention. The first stage involves data collection from various sources (Figure 2).

Photographs: Considered the easiest and most harmless source for facial information, photographs should be taken in color, with lateral and front-profile views, as well as 45° photographs from both sides. Both intraoral and extraoral photographs are necessary [27].

Radiographs: While 2D radiographs are an older method for visualizing bone structure and obtaining information about hard and soft tissue size and shape, they are inappropriate for the clinical planning of 3D structures [28].

3D Scanning: This method provides high-resolution data for texture. It can capture both intraoral and facial information. However, it has limitations, such as difficulty scanning black surfaces like hair, which are often ignored [29].

CT/CBCT/MRI Scanning: These procedures produce a series of planar images that can be compiled to create a three-dimensional object from digital data [30].

Segmentation and Visualization of the Virtual Digital Model

Anatomical properties in CBCT scans can be visualized using segmentation or surface rendering. Specific thresholds in Hounsfield/grayscale units are utilized to create differentiated surfaces. Edge detection algorithms allow the visualization of various organs, such as the upper jaw, providing several anatomical features [29].

Alignment and Integration

After data collection, the data is typically accessed in the form of three-dimensional point clouds. For example, 3D scans of the face include eye, lip color, skin, and texture information, while CBCT scans provide density information. These data sources must be aligned properly to prepare them for use in VSP [31].

Virtual Surgery Planning

Following the previous steps, the actual VSP begins. This step requires practice using all the planning and visualization tools encoded by the software, necessitating cooperation between bioengineers, clinical technicians, and medical practitioners. Proper diagnosis is the most crucial step in virtual planning. Analytical tools for 3D data measurement can detect defects, dysmorphologies, and deficiencies. Treatment planning can proceed once the differential diagnosis is established and plan parameters are calculated. Osteotomy location and angle are general craniofacial surgical plan parameters. Final alignment and virtual osteotomy can be simulated, though the virtual osteotomy is considered the weakest point of VSP due to its sensitivity and reliance on technician expertise. Therefore, predictive models are crucial to avoid this issue [29].

Manufacturing

Using digital data, 3D models can be printed to predict post-treatment outcomes. This process is essential when patient-specific tools are needed to facilitate the operative procedure. These tools can include surgical guides, such as osteotomy guides and screws, or patient models, typically manufactured using 3D printing directly or indirectly. Biocompatible materials are selected according to the specific application. Technicians plan the 3D printing parameters for each tool to be manufactured. Additionally, post-printing processes, such as chemical etching, can enhance osseointegration [2,29].

In-house 3D Printing

The preparation of digital files, including CBCT models and scans, is handled by in-house computer technicians who compile the necessary data for surgery. Aligning the CBCT images with the midsagittal reference plane obtained during clinical examination ensures accurate correlation with computer models. The jaws are then segmented according to the surgeon’s preferences, with osteotomies designed accordingly. The surgeon provides the computer technician with the intended surgical movements to simulate the operative plan. The surgeon then reviews, approves, and rejects this plan to achieve optimal outcomes [21]. Following this, final splints are 3D-printed, and cutting and fixation guides are fabricated if necessary, ensuring the surgeon executes the plan as intended. This collaborative approach between the surgeon and computer technician enhances efficiency, reducing the time required for planning from 6-8 hours to just 15-20 minutes. Additionally, it allows for better visualization of the final outcome in 3D, enabling the identification and resolution of potential issues before entering the operating room. Data collection has shown that this method can achieve surgical accuracy within 1 mm or 1 [21].

3D Printing in Orthognathic Surgery

Advancements in three-dimensional imaging technology, such as medical CT and CBCT scans, have provided more accurate anatomical descriptions than two-dimensional imaging, aiding clinicians in diagnosis, treatment planning, and predicting outcomes for orthognathic surgery. The CBCT technique allows for high-quality 3D reconstruction models, offering advantages over traditional CT imaging, such as shorter procedure times, lower radiation doses, and reduced costs [32]. This makes it a valuable tool for dentofacial imaging in orthognathic surgery [33]. By collecting CT data, natural visualizations of facial surfaces can be achieved using stereophotogrammetry and laser surface scanning [34,35]. Subsequently, virtual models of both the skin surface and CT-reconstructed osseous volume can be created to predict and simulate soft tissue outcomes [33,36]. Technological developments, including CBCT, intraoral digital scanning, three-dimensional stereolithographic printing (CAD/CAM; STL), and software enhancements, have revolutionized orthognathic surgical planning [37,38]. This combination of technology is collectively referred to as computer-aided surgical simulation (CASS) or computer-aided orthognathic surgery (CAOS) [39].

Comparison between VSP and TSP

Numerous studies have compared TSP and VSP, focusing on procedural accuracy and the accuracy of hard and soft tissue simulation [11,30,40,41].

Accuracy of the Procedure

VSP eliminates many laboratory steps and avoids errors associated with traditional procedures. The digital method makes planning faster and more repeatable [42]. Additionally, problems associated with 2D images, such as superimposition and magnification, are mitigated with 3D imaging [11].

Hard Tissue Accuracy

This literature review relies on randomized controlled studies to assess the accuracy of VSP compared with TSP, yielding varied results. In the study by De Riu et al., the percentage of angular and linear alignment of anatomical landmarks was evaluated in patients with facial asymmetry. VSP showed statistically significant accuracy in the mandibular sagittal plane, centering of the dental midline, and alignment of the lower interincisal point compared with TSP [38]. Bengtsson et al. found that hard tissue accuracy improved with VSP compared to TSP by comparing outcomes in the anteroposterior dimension for most cephalometric landmarks. VSP also improved outcomes in asymmetric facial appearance and malocclusion [40]. Another study by Schneider et al. compared the final surgical outcomes and planned positions using angular measurements, including the anteroposterior dimension [41]. The difference was statistically significant between VSP and TSP, with VSP improving the accuracy of outcomes [41]. According to a study by Hanafy et al., hard tissue accuracy was enhanced with VSP compared to TSP by analyzing surgical outcomes and assessing differences in the anterior, vertical, posterior, and mediolateral dimensions [43]. Conversely, Van Hemelen et al. did not find statistically significant differences in hard tissue accuracy between VSP and TSP when comparing the final surgical outcomes and planned positions using cephalometric landmarks [44]. Similar results were found in studies by Xu et al., which reported no difference in hard tissue accuracy between VSP and TSP [45].

In conclusion, the accuracy of hard tissue following orthognathic surgery using VSP compared to TSP remains contested. However, many studies have shown a statistically significant enhancement in hard tissue accuracy between the planned position and the final surgical outcome with VSP compared with TSP. Table 1 displays the characteristics and conclusions of the previous trials.

**Table 1:** Characteristics and conclusions of studies evaluating the accuracy of predicting hard tissue changes F/M: female/male; NA: not available; TSP: traditional surgical planning; VSP: virtual surgical planning; BSSO: bilateral sagittal split mandibular osteotomy; LFI: Le Fort I maxillary osteotomy; Bimax: bimaxillary osteotomy; HRQoL: patient’s health-related quality of life, TMD: temporomandibular disorders; TMJ: temporomandibular joint; CAD/CAM: computer-aided design and machining

Author & Study Design	Participants	Sample Size (F/M)	Age	Intervention Technique	Surgery (Involved jaws/specific surgery)	Software	Follow-up	Splint	Outcomes	Conclusion
Van Hemelen et al., 2015 [44], RCT	Patients with maxillofacial deformity	66 (37/29)	Mean 19.78 y	TSP: 35 VSP: 31	19 single-jaw surgery (11 BSSO; 6 BSSO + genioplasty; 2 LFI) 47 Bimax surgery (1 LFI + genioplasty; 25 Bimax; 21 Bimax + genioplasty)	TSP: NA VSP: Maxilim	Before-4 months after surgery	TSP: Manual VSP: CAD/CAM	Height and depth differences were determined by measuring the distances between corresponding landmarks in the sagittal plane, matching the anticipated preoperative and postoperative points.	The TSP approach and the VSP methodology are comparable in hard tissue planning. Soft tissue planning can be done more precisely by using the VSP approach.
Bengtsson et al., 2018 [40], RCT	Inclusion criteria are patients aged 18 to 30 diagnosed with Angle Class III occlusion and a minimum 5-mm negative horizontal overjet. Excluded are patients with TMD issues, substance misuse, severe psychiatric illnesses, or systemic musculoskeletal ailments.	57 (27/ 30)	18-28 (mean 20.8 y)	TSP: 29 VSP: 28	NA (LFI, segmented LFI, BSSO, Vertical ramus mandibular osteotomy, genioplasty)	TSP: Facad VSP: Simplant PRO 12.02 OMS	Before-12 months after surgery	TSP: Manual VSP: Manual	The study examined variations in cephalometric measurements at the 12-month follow-up, HRQoL, the link between cephalometric accuracy and patient-reported outcomes, changes in facial appearance, the relationship between HRQoL and cephalometric accuracy, and costs (monetary, temporal, and radiation dosage).	The study found that TSP and VSP approaches predict facial results with similar accuracy, and HRQoL improvements were evident regardless of the method. There was no significant difference between the two. Both methods improved facial aesthetics post-surgery and took the same time to complete, but VSP required higher radiation doses, while TSP was less expensive.
Schneider et al., 2019 [41], RCT	Inclusion criteria are healthy adults undergoing Bimax surgery for skeletal Class II malocclusion. Exclusion criteria include a history of hemifacial microsomia, craniosynostosis, degenerative or inflammatory conditions, or facial trauma.	21 (12/9)	23-52.1y (mean 31.1 y)	TSP: 12 VSP: 9	Bimax surgery (LFI, BSSO)	TSP: NA VSP: Dolphin 3D Imaging	Before-after surgery	TSP: Manual VSP: CAD/CAM	Difference between the planned and postoperative situation (SNA/SNB/ANB), the time required for surgery (min), and total costs of planning (€).	The operation takes noticeably less time and is more accurate when VSP technology is used in conjunction with printed acrylic splints, 3D models of the jaws, and pre-bent osteosynthesis.
Hanafy et al., 2020 [43], RCT	The patient’s consent to participate in the trial, completing the informed consent form, and not having any systemic conditions that could hinder bone repair or make them unfit for surgery were the requirements for inclusion. Previous major jaw surgery, cleft palate, or active symptoms of TMJ dysfunction were considered exclusion criteria.	18 (9/9)	19-24y (mean 21.22)	TSP: 9 VSP: 9	Bimax surgery	TSP: NA VSP: 3-matic 11.0; Materialise NV	4 months after the procedure	TSP: Manual VSP: CAD/CAM	Angular measurements. Wound healing, deviation from plan, planning time, operative time	With the use of this innovative technology, situations involving skeletal asymmetry were made easier, operating hours were shortened, and a trainee surgeon was able to complete the treatment quickly and accurately. The primary impediment was the expensive price.
Xu et al., 2020 [45], RCT	Criteria included Bimax surgery (LFI and BSSO), nose severe asymmetry (less than 4 mm mandibular deviation and 4° occlusal cant), absence of TMD or craniofacial abnormalities, availability of CT scans for preoperative, postoperative, and one-year follow-up, and the ability to use virtually designed and printed occlusal splints.	30 (20/10)	Mean 25y	TSP: 15 VSP: 15	Bimax surgery (LFI osteotomy and BSSO);	TSP: NA VSP: into Mimics (v16.0, Materialise)	1 year	TSP: Manual VSP: fabricated by a prototyping machine (DentLab One, SHINING 3D).	The planned and follow-up outcomes, as well as the planned and actual results, were compared to determine the absolute linear differences.	No discernible difference exists between TSP and VSP when using an intermediate splint. Errors in Bimax surgery may stem from planning transfer with these splints. New positioning devices could enhance the accuracy of VSP over TSP.

Soft Tissue Accuracy

Prediction and evaluation of orthodontic and surgical results include soft tissue and photographic three-dimensional images [7]. Simulating soft tissues’ reactions to planned surgical movements can be a valuable tool for patient communication and discussing treatment options [46]. According to Chen et al., soft tissue accuracy improved with the VSP technique compared with TSP [47]. However, De Riu et al. found no statistically significant difference in the rate of soft tissue alignment (soft tissue menton) to the facial midline between VSP and TSP [48]. As a result, approximate outcomes can be predicted for soft tissue. Although VSP can be useful for predicting postsurgical outcomes, it has lower accuracy than bone predictions. Additionally, soft tissue remodeling issues may occur following hard tissue changes [49]. Table 2 displays the characteristics and conclusions of the previous studies.

**Table 2:** Characteristics and conclusions of studies evaluating the accuracy of predicting soft tissue changes F/M: female/male; NA: not available; TSP: traditional surgical planning; VSP: virtual surgical planning; BSSO: bilateral sagittal split mandibular osteotomy; LFI: Le Fort I maxillary osteotomy; Bimax: bimaxillary osteotomy; CROS: conventional resin occlusal splint group; DOS: digital occlusal splint group; DT: digital templates; CAD/CAM: computer-aided design and machining

Author & Study Design	Participants	Sample Size (F/M)	Age	Intervention technique	Surgery (involved jaws /Specific surgery)	Software	Follow-up	Splint	Outcomes	Conclusion
De Riu et al., 2014 [48], RCT	Inclusion criteria: occlusal plane cant >3° or midline discrepancies >2.5 mm; all central incisors present; the presence of pre- and postoperative radiographs with plaster casts in the classic group and cranial CT images with digital photographs in the 3D-based digital group. Exclusion criteria: previous facial trauma, functional mandibular deviation, or incomplete records.	20 (10/10)	21-54 Y	TSP: 10 VSP: 10	20 Bimax surgery (Osteotomies on the maxilla, mandible, and genioplasty)	TSP: NA VSP: Maxilim	Before-after surgery	TSP: Manual VSP: CAD/CAM	Distances between upper and lower interincisal points, skeletal and soft tissue menton, and the ideal facial midline; distances between the maxillary sagittal plane, mandibular sagittal plane, and ideal midsagittal plane.	Compared with the TSP method, the adoption of VSP for facial asymmetry allowed better control and accuracy in repositioning and alignment of the maxilla and the mandible.
Elshebiny et al., 2019 [46], Cohort study	Exclusion criteria included patients with craniofacial anomalies and patients with missing records.	20 (11/9)	22.7 y		LF1 movement with BSSO with or without a genioplasty	VSP: Dolphin three-dimensional (3D) software version 11.9	At least 6 to 12 months postoperative	NA	ANS in sagittal and vertical direction. Occlusal Plane (°). B Point in Sagittal direction.	Dolphin 3D software struggles to predict midface soft tissue changes after double-jaw surgery but accurately simulates upper and lower facial thirds. Clinicians should carefully inspect these programs for errors.
Chen et al., 2020 [47], RCT	Patients aged 18-40 with dentomaxillofacial deformities requiring Bimax surgery were included. Exclusions were cleft lip and palate or craniofacial syndrome, deformities from trauma, tumor, or iatrogenic factors, previous orthognathic surgery, and planned segmental LFI osteotomy.	60	18-40 y	CROS: 20. DOS: 20 DT: 20	Bimax surgery	CROS: NA DOS: Mimics 19.0 DT: 3-Matic	7 days postoperative	CROS: Manual. DOS: fabricated by a 3D printer. DT: fabricated by a 3D printer.	The primary outcome measure for this study was the mean distance between the planned and actual postoperative positions of eight selected points in the upper jaw.	Thorough diagnosis, sensible planning, and precise operation are crucial for orthognathic surgery success. This study showed that printed cutting and repositioning templates transfer the maxillary surgical plan to the operating room with superior accuracy compared to traditional or digital occlusal splints, without significantly increasing operating time.

Accuracy of the Surgery

3D planning offers several advantages by providing detailed anatomical information about each patient [50]. VSP creates accurate surgical plans that assist clinicians during operations, helping to determine osteotomy lines and review surgical options, thereby producing more accurate outcomes compared to TSP [11]. When comparing VSP with TSP, results show similar hard tissue accuracy in the sagittal plane. However, VSP is more accurate in the anterior area of the maxilla. Both techniques exhibit better accuracy for the maxilla than the mandible. The coronal plane accuracy is a notable disadvantage for TSP, making VSP a more reliable technique for cases of facial asymmetry [7]. VSP in orthognathic surgery is effective and accurate, as evidenced by post-treatment and pre-treatment comparisons using cephalometric measurements [51]. VSP also considers growth and the possibility of relapse, creating multiple scenarios to achieve the best outcomes. Consequently, VSP enhances accuracy, reduces complications, and minimizes the need for reoperations [7].

## Conclusions

Recently, surgical procedures for the correction of dentofacial disorders have become more widely performed. TSP in orthognathic surgery has limitations due to the use of 2D radiographs and dental models for diagnosis and planning. With the recent advancements in 3D printing technology in medical fields, VSP now includes 3D imaging, CAD/CAM techniques, image guidance technology, and computer simulation software. These techniques help create patient-specific devices based on three-dimensional models for orthognathic surgery, enhancing the accuracy of treatment planning and the precision of surgical procedures.

Numerous studies have indicated that 3D printing helps surgeons shorten operative times, improve the predictability of surgical outcomes, and increase the safety of surgical processes. It also reduces surgical difficulties by allowing clinicians to detect problems and modify surgical plans before operations. Additionally, it minimizes human errors and accurately translates VSP into real surgical operations.
